# A Leap toward Quasi-Solid-State Chloride-Ion Batteries with Metal–Organic Frameworks

**DOI:** 10.1021/acsenergylett.5c02431

**Published:** 2025-12-16

**Authors:** Valentino G. Martello, Alessandro Piovano, Matteo Bonomo, Mircea Dincă, Silvia Bordiga, Claudio Gerbaldi

**Affiliations:** † University School for Advanced Studies, IUSS Pavia, Palazzo del Broletto, Piazza della Vittoria 15, I-27100, Pavia, Italy; ‡ University of Torino, Department of Chemistry, 9314 NIS Interdepartmental Centre and INSTM Reference Centre, Via Quarello 15a, 10135, Turin, Italy; § GAME Lab, Department of Applied Science and Technology, 19032 Politecnico di Torino, Corso Duca degli Abruzzi, 24, 10129, Torino, Italy; ∥ National Reference Centre for Electrochemical Energy Storage (GISEL)−INSTM, Via Giusti 9, 50121, Firenze, Italy; ⊥ Department of Basic and Applied Sciences for Engineering (SBAI), Sapienza, University of Rome, Via Del Castro Laurenziano 7, 00161, Roma, Italy; # Department of Chemistry and Department of Chemical Engineering, Massachusetts Institute of Technology, Cambridge, Massachusetts 02139, United States

## Abstract

The development of solid-state electrolytes is a major focus in energy storage, offering improvements in both safety and performance. In chloride-ion batteries (CIBs), where electrode dissolution in liquid electrolytes remains a critical challenge, solid-state alternatives are especially attractive. Herein, we demonstrate for the first time the suitability of a metal–organic framework (MOF) as a quasi-solid-state single-ion electrolyte for CIBs. The cationic Al-based MOF MIP-213 ([Al_18_(μ_2_-OH)_24_(OH_2_)_12_(mdip)_6_]­6Cl·6H_2_O) exhibits a chloride ion conductivity of 1.1 × 10^–6^ S cm^–1^ at 25 °C, is nonflammable, electrochemically stable up to 4.2 V vs Li^+^/Li, and enables single-ion transport in a Li|MIP-213|FeOCl full cell over 100 cycles with Coulombic efficiency > 90%, while maintaining structural integrity. Although further optimization of the CIB components will be needed to enable cycling at higher current regimes and approach practical application, these findings establish MOFs as promising platforms for designing stable and efficient quasi-solid-state batteries.

## Graphical abstract

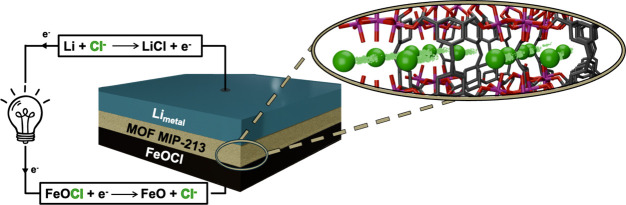

The leading technology utilized for energy storage is Li-ion batteries (LIBs), in which liquid electrolytes are dominant. For decades, solid-state energy storage has been regarded as the next frontier, targeting comparable performance and higher safety.^1,2^ Driven by the desire of addressing issues such as resource scarcity and high costs, researchers have increasingly explored alternative systems to LIBs. These include those based on metal cations such as Na^+^, Mg^2+^, Zn^2+^, and Al^3+^,^3–9^ as well as emerging technologies that shift away from the conventional cation-shuttling mechanism. Notably, systems where halide anions play the active transport role, such as in chloride-ion and fluoride-ion batteries (coded as CIBs and FIBs, respectively), are also gaining attention.^10–12^ In particular, interest in CIBs has grown over the past decade due to the abundance and affordability of the materials used in cell assembly, their promising performance, high safety and low toxicity (*i.e.* anyhazardous elements involved).^13,14^ For instance, iron chlorides proposed as cathode materials for CIBs cost merely 1–2% of traditional cathode materials used in LIBs.^15^ Additionally, CIBs are characterized by the absence of metal plating on the anode, which eliminates the risk of dendrite formation (and of possible short-circuit failures), as the charge transport mechanism solely relies on chloride ion shuttling.^10^ Furthermore, the theoretical volumetric energy density of CIBs exceeds 2500 Wh L^–1^, three times that of Li-ion cells. Although this value is even higher for FIBs (>4800 Wh L^–1^), CIBs remain the preferred choice among the two due to easier handling of materials and lower environmental impact.^16–18^

A CIB was reported for the first time by Zhao *et al.* in 2014, employing Li metal as anode, an ionic liquid as electrolyte and a mixture of metal chlorides as the cathode.^19^ They demonstrated that the concept is viable, also evidencing some challenges, chief among them being the partial dissolution of the cathode in the electrolyte. This solubility-related issue is also a common limit for many metal anodes based on abundant elements like Na, Ca and Mg.^20,21^ Therefore, transitioning toward solid-state electrolytes or quasi-solid-state electrolytes (QSSEs) constitutes a valuable solution to the mentioned problems. However, most (Q)­SSEs explored so far in this context achieve reasonable ionic conductivity of >10^–5^ S cm^–1^ only above 150 °C, with perovskites making some progress in this field more recently (benchmark list in reports most significant and recent results for (Q)­SSEs).^11,22–30^

One possible approach to designing QSSEs for secondary batteries is the use of metal–organic frameworks (MOFs).^31^ While MOFs offer porous and tunable architectures for ion transport, in the majority of reported battery-relevant systems, appreciable conductivity arises only in the presence of confined guest molecules (*e.g.*, carbonate solvents or ionic liquids), which mediate vehicle-assisted transport or solvent-assisted hopping. Dry frameworks typically exhibit much lower conductivity. Consistent with this, Hou *et al.* identified solvent-assisted hopping as the dominant pathway in a MOF-based QSSE.^32–34^ At the same time, Deriche *et al.* caution that extrinsic proton/solvent contributions can inflate apparent conductivities and recommend rigorously anhydrous controls to isolate any intrinsic framework-mediated transport.^35^ In this context, when conduction relies on confined guests, the quasi-solid-state electrolyte (QSSE) designation is the most appropriate.

Building on this, the remarkable structural and compositional versatility of MOFs has driven growing interest in their use not only as QSSEs but also as electrode materials across a variety of battery chemistries, including Li^+^, Na^+^, and Mg^2+^ systems.^36–40^ An especially appealing property that MOFs can exhibit as QSSEs is single-ion conduction, a mechanism in which only the electrochemically active ions contribute to ionic transport. This feature helps mitigate concentration polarization effects, which typically arise when the counterion migrates in the direction opposite to battery polarity under high current loads.^41^ To enable this, a MOF could function as a (quasi)­solid-state single-ion electrolyte for CIBs if it had a positively charged framework or static positively charged functional groups, thereby hosting free charge-balancing anions within its pores.^37,40^

Herein, we show that a cationic MOF with Cl^–^ counterions in its pores, MIP-213­(Al) ([Al_18_(μ_2_-OH)_24_(OH_2_)_12_(mdip)_6_]­6Cl·6H_2_O) constructed using the ligand 5,5′-methylenediisophthalic acid (H_4_mdip), functions as a single-ion chloride electrolyte, representing the first example of such a function for a MOF. This material was initially developed for CO_2_ capture,^42^ and its structural features make it particularly suited for ion transport in solid-state devices. Importantly, its synthesis involves only relatively affordable materials and is conducted in an alcohol–water mixture. A further notable advantage from a practical viewpoint lies in the incorporation of chloride anions directly from the AlCl_3_ precursor, eliminating the need for postsynthetic anion exchange or modification.

Motivated by this potential, we optimized the synthetic procedure (details in , Figures S1–S5) and measured the ionic conductivity of MIP-213 to evaluate its feasibility as a quasi-solid-state electrolyte in CIBs. The results are noteworthy, revealing relatively high ionic conductivity values on the order of 10^–6^ S cm^–1^ at 25 °C (Figure 1a). The ionic conductivity was measured by electrochemical impedance spectroscopy (EIS) in a SS|MIP-213|SS cell configuration (where SS stands for stainless steel) as a function of temperature and plotted vs 1000/T (Figure 1a, impedance spectra in , fitting parameters in ). Since MIP-213, like most MOFs, has not been observed to conduct ions under dry conditions, different aliquots of propylene carbonate (PC) were added to the powder before cell assembly, to enable ionic conduction (). Notably, when pressed into a pellet at the moderate stack pressure of ∼30 MPa (other values explored are reported in ) with an MOF:PC ratio of 10:4, MIP-213 shows a low activation energy value of 0.18 eV (similarly, the others are in the range 0.17–0.20 eV), which is indicative of efficient ion conduction and enhanced mobility of charge carriers.

**Figure 1:**
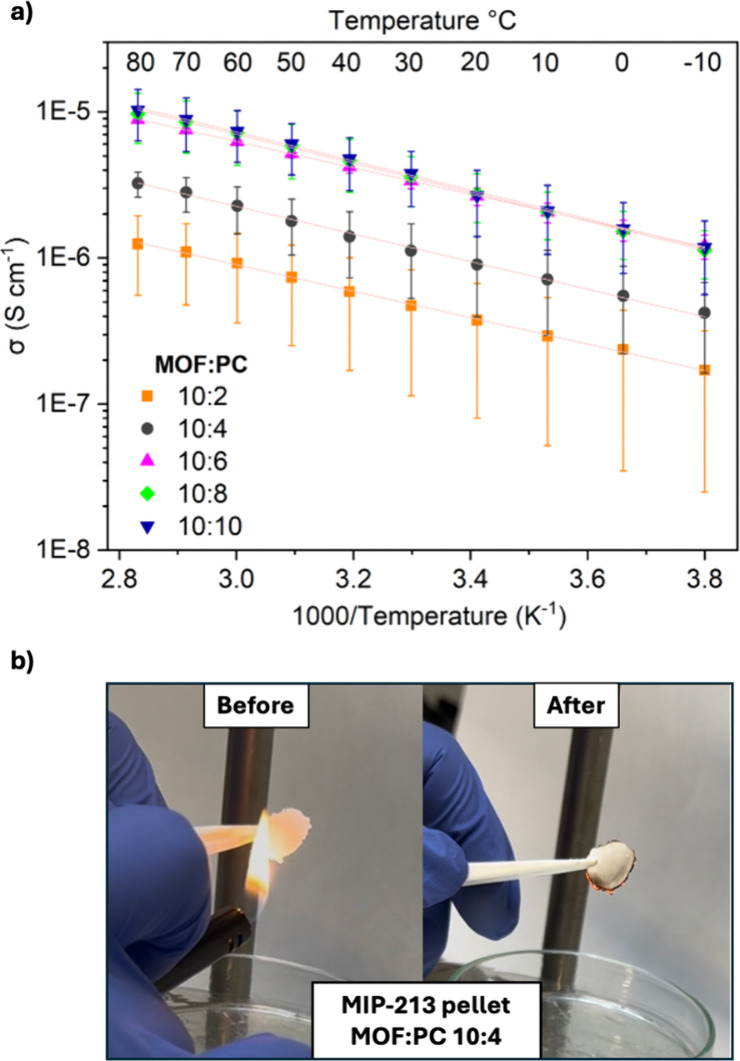
a) Ionic conductivity versus 1000/T plot of MIP-213 measured with different solvent (PC) aliquots up to MOF saturation. The fit lines are displayed in light red (R^2^ > 0.999 for all), and the error bars represent the standard deviation across four independent measurements on separately prepared pellets. The apparent increase in error with decreasing PC content is due to the logarithmic scale. b) Flame test of a MIP-213 pellet with MOF:PC ratio of before and after 1 s of flame exposure.

A direct correlation between solvent content and ionic conductivity is observed: beyond a MOF:PC ratio of 10:6, the conductivity plateaus, likely due to complete pore saturation. This suggests that the most effective transport pathway is enabled below this threshold, while further solvent likely accumulates at particle interfaces without substantially enhancing the ion mobility. Although these data are consistent with a key role of solvent confined within the framework and considering that chloride counterions are structurally required to stabilize the cationic lattice and are therefore less likely to migrate extensively outside the pores, we cannot exclude contributions from interfacial solvent even at lower loadings. Consequently, all subsequent electrochemical experiments were carried out using a MOF:PC ratio of 10:4, which we found as optimal for high conductivity (1.1 × 10^–6^ S cm^–1^ at 25 °C), while maintaining the MOF’s free-flowing powder behavior (). Indeed, higher PC loads afford sticky agglomerates and eventually form a paste. Notably, the ratio MOF:PC 10:4 corresponds to a total amount of solvent in the electrochemical cell of 6.5 μL cm^–2^, which is about 1/20 of the amount typically used in cells based on liquid electrolytes, thereby ensuring significantly enhanced fire resistance (as proved by the flame exposure test shown in Figure 1b and further discussed in detail in ), substantially improving the safety aspects of the final device.

To elucidate the ion transport dynamics in these minimally solvated quasi-solid-state systems, it is important to consider both hybrid vehicular diffusion within the MOF pores and cooperative Grotthuss diffusion along hydrogen-bond networks outside the pores.^40^ It is reasonable to assume that both diffusion processes play a role in our system, eventually leading to a solvent-assisted hopping,^32^ given that the secondary building unit layers (consisting of chains of AlO_4_(OH)_2_ and AlO_2_(OH)_3_(H_2_O) octahedra) are interconnected by tetracarboxylate linkers forming a three-dimensional cationic framework whose charge is balanced by chloride ions. The latter are found within pores and exhibit H-bonding interactions with structural water molecules pertaining to the AlO_2_(OH)_3_(H_2_O) octahedra ().^42^ As such, Cl^–^ ions could hop through H-bonds and diffuse into the cavities.

To prepare the MOF for chloride ion conduction, solvent molecules occupying the pores of the as-synthesized MOF must be removed and replaced with PC. The thermogravimetric analysis of MIP-213 () reveals a continuous weight loss involving different processes from RT up to complete degradation at 500 °C. To better understand the thermal evolution of the material and identify a suitable pretreatment procedure (*i.e.*, activation), in situ diffuse reflectance infrared Fourier transform (DRIFT) spectroscopy was carried out under dynamic vacuum at increasing temperatures and complemented with powder X-ray diffraction (PXRD). This was conducted over a range from room temperature (RT, 25 °C) to 280 °C: above this upper limit MIP-213 was observed to severely degrade, even though major broadenings of the PXRD peaks are already noticeable at 220 °C (Figure 2a).

**Figure 2:**
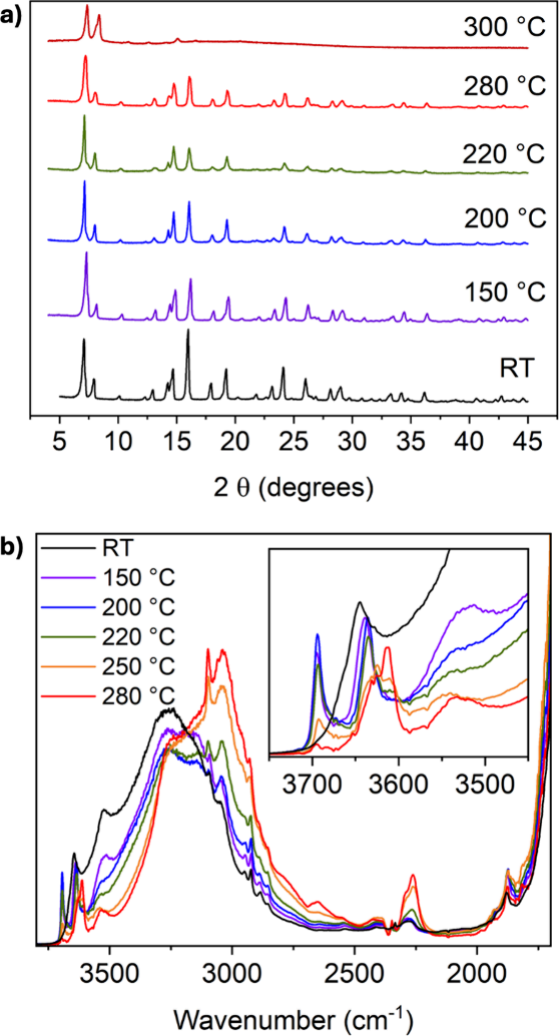
a) PXRD patterns measured in the temperature range RT–300 °C after 2 h thermal treatments. b) DRIFT-IR spectra collected at different temperatures under vacuum (all the spectra are reported after reaching equilibrium at the specified temperature) between RT and 280 °C.

The DRIFT spectrum collected at RT (Figure 2b, black curve) presents a broad absorption band centered at ∼3250 cm^–1^ with a shoulder at 3520 cm^–1^, and a sharp band at 3645 cm^–1^. Below 1850 cm^–1^ the spectrum is dominated by the out-of-scale absorption of the vibrational modes of the framework. The broad absorption band can be attributed to ν­(OH) from structural H_2_O, physisorbed water, and benzyl alcohol (from the synthesis), involved in H-bonding interactions with each other and with chloride ions. Overlapped with this broad band, at around 3000 cm^–1^ some weak and sharp bands are visible, likely corresponding to aromatic ν­(CH_*x*_) from the organic linker. Upon outgassing at higher temperature (purple curve, 150 °C), the broad band slightly decreases in intensity, testifying to removal of physisorbed molecules (structural OH in interaction with Cl- ions is still visible, ), while the emergence of two additional sharp bands at 3694 cm^–1^ and 3638 cm^–1^ is noticed. These fall in the range of isolated OH groups, detectable at this temperature, since they are no longer involved in interactions with physisorbed species.^43^ Further increasing the temperature to 200 °C, which according to TGA should cause the removal of solvent molecules (), does not significantly alter the main IR features of the MOF.

Further raising the temperature does evidence the onset of chemical transformations: the bands corresponding to isolated OH groups (at 3694 and 3638 cm^–1^) gradually decrease in intensity between 220 and 280 °C. A concurrent increase of a broad absorption band centered at ∼3100 cm^–1^ can be attributed to the formation of HCl trapped in the pores of the material and involved in H-bonds with the functional groups in their surroundings.^44^ Simultaneously, a new band at ∼2250 cm^–1^ appears and grows in intensity. It is attributable to the stretching of physisorbed CO_2_, likely trapped in the material after the decarboxylation of the linker. These observations, supported by PXRD data, indicate the onset of MOF degradation above 200 °C, likely due to dehydroxylation of the metal cluster and linker deterioration. Based on these considerations, 200 °C was selected as the activation temperature for the MOF to remove residual solvent molecules from the synthesis, prior to introducing PC into the cavities. The activation of the MOF was evaluated by a CO_2_ sorption volumetric analysis. CO_2_ was preferred over N_2_, given that, due to its larger kinetic radius, molecular nitrogen is prevented to access the pores already hindered by the presence of Cl^–^. In line with the previously reported value, MIP-213 showed a CO_2_ uptake of ∼1.4 mmol g^–1^ at 0 °C ().^42^

The activated MOF was characterized electrochemically with linear sweep voltammetry (LSV, in Figure 3a) in a Li|MIP-213|Ni-CC (Ni-CC stands for carbon-coated nickel current collector) cell configuration at 25 °C, which confirmed it to be stable up to 4.2 V vs Li^+^/Li, and hence compatible with most of the cathode materials proposed for CIBs such as FeOCl (demonstrated in this work as a proof of concept), but also BiOCl, Sb-based compounds, layered double hydroxide (LHDs) and metal chlorides (which are typically discarded because of the high solubility in liquid electrolyte, but which in this case could also be explored given the quasi-solid state nature of the electrolyte).^11,14,45^

**Figure 3:**
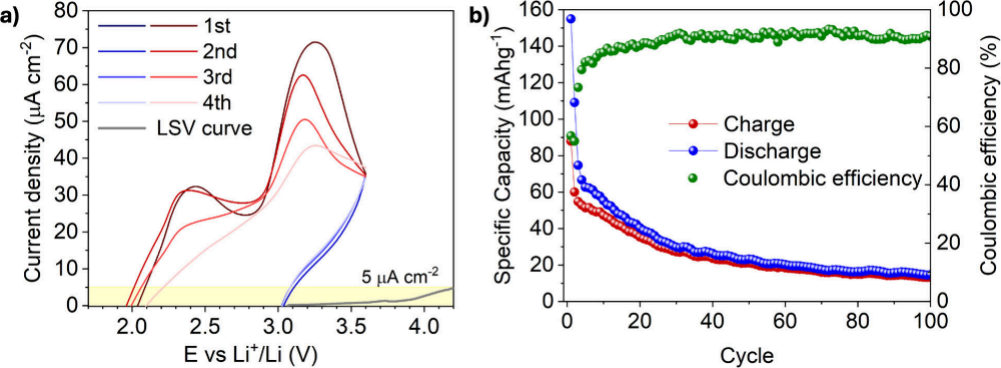
a) CV-LSV graphs combined. Cell configurations Li|MIP-213|FeOCl (oxidation curves in red and reduction in blue) and Li|MIP-213|Ni-CC (gray curve, with the indication of the current limit of 5 μA cm^–2^ for the commercialization of new electrolytes^46^) were employed, respectively. b) Galvanostatic charge/discharge cycling of the cell Li|MIP-213|FeOCl at 25 °C, where MIP-213 is used as a pellet with an MOF:PC ratio of 10:4. Current applied: 15 mA g^–1^ (theoretical C/20 regime, meant to establish this proof-of-concept cycling).

Indeed, the LSV graph shows that no oxidation peaks attributable to MIP-213 electrolyte are present within the potential range of 1.5–3.6 V vs Li^+^/Li adopted for the cyclic voltammetry (CV) of the solid-state cell assembled with iron oxychloride as cathode material (FeOCl, PXRD patterns in ). In detail, the smooth CV profile (partially shown in Figure 3a and fully reported in ) shows two different processes of oxidation at ∼2.40 and ∼3.25 V with respective reduction peaks at ∼1.80 and ∼2.55 V, which can be ascribed to the FeOCl/FeO conversion, alongside minor FeOCl_*x*_ intercalation reactions.^47^ These results align closely with those of FeOCl reported in the literature,^48–51^ showing no additional features from side redox reactions of the MOF-PC composite.

Here, FeOCl was chosen as the cathode material for its readiness of preparation (thermal decomposition of FeCl_3_·6H_2_O), low cost, and low environmental impact. Moreover, conversion type metal oxychlorides like FeOCl are the most studied cathode materials for CIBs, followed by insertion/intercalation type layered double hydroxide (LHDs) that drew some interest in recent years.^10^ Specifically, FeOCl has already been used for CIBs, in combination for instance with ionic liquids or poly­(ethylene oxide) (PEO) and chloride salts as electrolytes,^27,47,49^ showing, in the best case, a maximum discharge capacity of ∼160 mAh g^–1^ and enhanced stability against dissolution if compared to simple metal chlorides. Nonetheless, all literature reports employing pristine FeOCl show a capacity drop after a few cycles caused by the low electrical conductivity of iron oxychloride and the large volume change in the phase transformation during cycling,^47^ which indicates that the observed capacity fading is intrinsic to FeOCl and not related to the MOF electrolyte. Interestingly, it has been demonstrated that this issue can be substantially mitigated by nanoconfining FeOCl particles within mesoporous carbon matrices.^50,51^ However, in the present study, we deliberately employed pristine FeOCl in order to isolate the role of the MOF-based electrolyte without introducing additional cathode modifications.

From the mechanistic point of view, Chen et al. demonstrated through TEM and XPS experiments that the redox activity of the FeOCl cathode is primarily attributable to a conversion reaction forming amorphous FeO (undetectable by PXRD), rather than to an intercalation mechanism, following the reaction FeOCl+Li → FeO+LiCl.^27^

In this work, the solid-state Li|MIP-213|FeOCl cell configuration resulted in stable galvanostatic cycling, delivering ∼60 mAh g^–1^ of reversible discharge capacity for around 20 cycles with good Coulombic efficiency (>90%). This performance is achieved despite an initially high irreversible capacity (∼155 mAh g^–1^), attributed to the cathode conversion process confirmed by post-mortem PXRD analysis (). Upon extended cycling, the cell still retains ∼20 mAh g^–1^ after 100 cycles, demonstrating promising long-term stability. This is to date the highest number of cycles achieved for a FeOCl cathode in a quasi-solid-state cell architecture (Figure 3b), and it is particularly notable because it can be achieved at room temperature. In addition, the voltage profiles are in accordance with the CV and literature data,^27,49,51^ and, despite the evident initial capacity drop, s-type profiles typical of the cathode materials are maintained ().

Interestingly, the comparison between the PXRD patterns of the pristine MOF and those recorded after cycling evidences remarkable structural stability of the material (Figure 4a). In fact, MIP-213 remains intact, and the particle morphology is well preserved, as confirmed by SEM analysis (Figure 4b) and EDX mapping (). Besides, EDX analysis detected no Fe signal, indicating that any cathode dissolution, if occurring, is negligible. This result stands out in comparison with other electrolytes for CIBs (mostly liquids or gel polymers), where the electrolytes eventually degrade, leading to thermal runaway, or dissolve the electrodes.^10–12,52^

**Figure 4:**
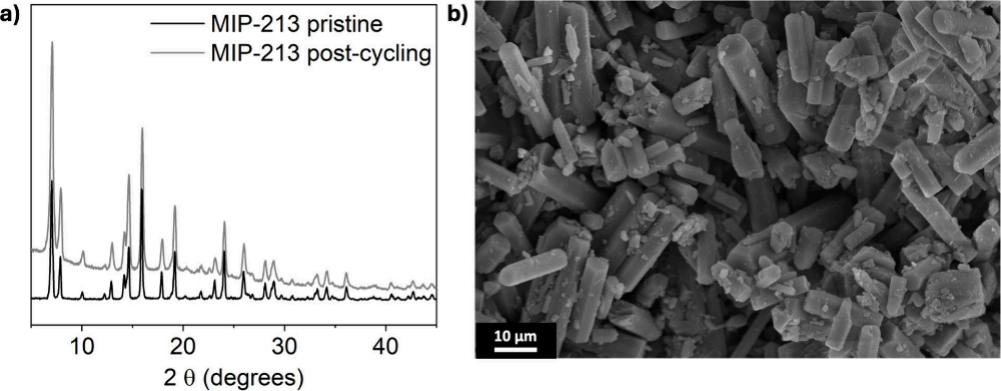
a) PXRD patterns of MIP-213 before and after cycling. b) SEM image of MIP-213 after cycling.

To conclude, while the absence of H^+^/OH^–^ signatures in the CV over the explored voltage range (1.5–3.6 V vs Li^+^/Li) is expected, as we are outside the proper evaluation window for protonic activity, the presented results are consistent with chloride-coupled behavior under our operating conditions. Specifically, the stable galvanostatic cycling and the preserved integrity of the current collector after cycling are inconsistent with extensive OH^–^ or protonic conduction within the intrinsically cationic MOF. By contrast, when electrode preparation was attempted in air, we observed pitting corrosion of Ni consistent with HCl evolution from FeOCl hydrolysis (). Taken together, these observations support chloride-dominated operation in our cells.

In summary, we report a comprehensive characterization and electrochemical investigation of MIP-213, a cationic MOF that functions as a single-ion solid-state electrolyte for chloride-ion batteries, with an anionic conductivity of 10^–6^ S cm^–1^ at 25 °C. The material enables the assembly of a full cell using lithium metal and iron oxychloride (FeOCl) as the negative and positive electrode, respectively. The cell exhibited a reversible capacity of around 20 mAh g^–1^ over 100 cycles with good Coulombic efficiency. MIP-213 showed exceptional stability versus electrochemical galvanostatic charge/discharge cycling, with the structure and the morphology of the material unchanged after operation.

Overall, this study establishes MIP-213 not only as the first solid-state and first single-ion MOF electrolyte for chloride conduction but also, more broadly, as one of the most effective QSSEs for CIBs, simultaneously addressing the long-standing challenges related to dissolution of electrode materials and high-temperature operating regime. These results highlight the feasibility of leveraging MOFs in next-generation energy storage for the design of more sustainable and safer (quasi)­solid-state rechargeable batteries.

## Supplementary Material





## Data Availability

All data for this article are available at Zenodo with the identifier 10.5281/zenodo.15429774. Some data supporting this article have been included as part of the .

## Notes

SEM, EDX, TGA, BET, ^1^H NMR, PXRD, structure visuals, and photos (PDF)
Videos (ZIP)
