# Supplementary material for: A Leap toward Quasi-Solid-State Chloride-Ion Batteries with Metal–Organic Frameworks
Source: ACS Energy Lett. 2025 Dec 16;11(1):419–25. doi: 10.1021/acsenergylett.5c02431 (PMC12797858; doi:10.1021/acsenergylett.5c02431)
Supplement: Supplementary file 1 [file nz5c02431_si_001.pdf]

A leap towards quasi-solid-state chloride-ion batteries with metal-organic frameworks

Valentino G. Martello,[a,b,d¶] Alessandro Piovano,[c,d] Matteo Bonomo,[b,d,e] Mircea Dincă,[f,] Silvia Bordiga,[b,*] Claudio Gerbaldi [c,d,*]*

[a] University School for Advanced Studies, IUSS Pavia, Palazzo del Broletto, Piazza della Vittoria 15, I-27100, Pavia, Italy

[b] University of Torino, Department of Chemistry, NIS Interdepartmental Centre and INSTM Reference Centre, Via Quarello 15a, 10135, Turin, Italy

[c] GAME Lab, Department of Applied Science and Technology, Politecnico di Torino, Corso Duca degli Abruzzi, 24, 10129, Torino, Italy.

[d] National Reference Centre for Electrochemical Energy Storage (GISEL)–INSTM, Via Giusti 9, 50121 Firenze, Italy

[e] Department of Basic and Applied Sciences for Engineering (SBAI), Via Del Castro Laurenziano 7, 00161, Roma, Italy

[f,#] Department of Chemistry and Department of Chemical Engineering, Massachusetts Institute of Technology, Cambridge, Massachusetts 02139, United States

Corresponding Author

***Mircea Dincă** – Department of Chemistry and Department of Chemical Engineering, Massachusetts Institute of Technology, Cambridge, Massachusetts 02139, United States; orcid.org/0000-0002-1262-1264; Email: mdinca@mit.edu.

***Silvia Bordiga** – University of Torino, Department of Chemistry, NIS Interdepartmental Centre and INSTM Reference Centre, Via Quarello 15a, 10135, Turin, Italy; orcid.org/0000-0003-2371-4156; Email: silvia.bordiga@unito.it.

***Claudio Gerbaldi** – GAME Lab, Department of Applied Science and Technology, Politecnico di Torino, Corso Duca degli Abruzzi, 24, 10129, Torino, Italy; orcid.org/0000-0002-8084-0143; Email: claudio.gerbaldi@polito.it.

Supporting Information

Table of Contents

Materials and Methods *2*
Materials *2*
Synthesis of MIP-213 .... *2*
Synthesis of FeOCl and preparation of FeOCl electrodes *7*
Methods *8*
Textural and physical chemical characterization of MIP-213 .... *8*
Ionic conductivity *8*
Electrochemical characterization *14*
Additional Results and Discussion *16*

Materials and Methods

Materials

5,5'-methylenediisophthalic acid (H_4mdip) was purchased from Ambeed. Aluminum chloride hexahydrate ($AlCl_3 \times 6H_2O$), iron chloride hexahydrate ($FeCl_3 \times 6H_2O$), anhydrous N-Methyl-2-pyrrolidone (NMP), lithium metal foil (200 µm), ethylene carbonate, dimethyl carbonate, benzyl alcohol, sodium hydroxide and ethanol were purchased at ACS reagent grade from Sigma Aldrich (Merck). Propylene carbonate battery grade was supplied by Solvionic (Toulouse, France). Carbon additive (C65 from Imerys, formerly Timcal), and polyvinylidene fluoride (PVdF, Solef 5130 from Solvay) binder were used as received.

Synthesis of MIP-213

We started from the reported synthetic approach.[1] However in our case, the harsh conditions (elevated temperature and basicity) degraded the borosilicate glass of the round bottom flask making silicon available in the reaction mixture and yielding sodalite ($Na_8(Al_6Si_6O_{24})Cl_2$, details

in **Figure S1**) instead of the targeted MOF. This can explain the presence of both Si and Na in the sample as detected by EDX analysis.

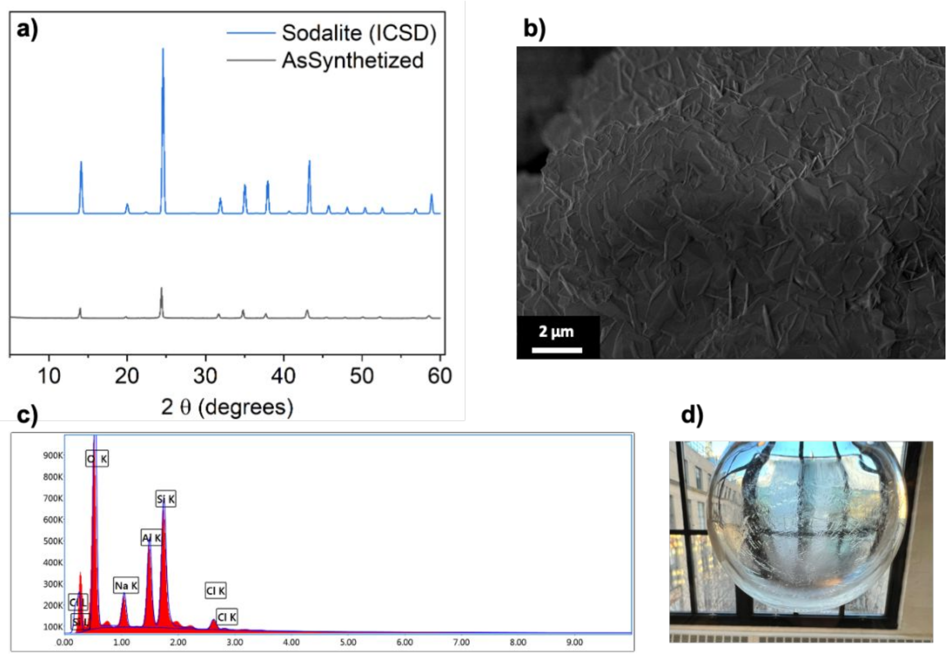

Figure S1. a) PXRD of the compounds obtained following the reported synthetic procedure for MIP-213 (black) compared with ICSD data for sodalite (blue); b) SEM image of the sample; c) EDX spectrum reporting the unexpected presence of Si and Na; d) the round bottom flask used for some trials of the synthesis. It shows defects in the glass which were not originally present.

Thus, we adopted a different synthetic pathway, obtaining MIP-213 via solvothermal method: the amount of 177 mg of $AlCl_3 \times 6H_2O$ (0.73 mmol) were dispersed in 4.40 mL of benzyl alcohol in a 23 mL Teflon vessel of an autoclave (Parr instruments model 4749). The linker, H_4mdip 77 mg (0.22 mmol), was first dispersed in 1.80 mL of water (pale yellow dispersion) and then solubilized upon addition of 0.44 mL 2M NaOH aq. solution, obtaining a clear red solution upon deprotonation of the linker. This mixture was added to the first dispersion in the autoclave, which was sealed and heated at 170 °C for 3 days (**Figure S2**). The reaction mixture was firstly centrifuged (2 × EtOH, 5000 rpm, 5 mins, 25 °C) and subsequently washed overnight using a

soxhlet extractor with ethanol as solvent. The off-white MOF was eventually dried at 70 °C overnight (yield: ~104 mg, ~81% considering the mmol of reactants and MIP-213 molar mass from the expected stoichiometry: 3496.56 g/mol).[6] **Figure S3**, **Figure S4** and **Figure S5** show the PXRD pattern of the structure, the SEM images along with the EDX map of MIP-213 and the ^{1}H-NMR collected after digestion of the MOF respectively. The synthesis has been up-scaled consistently to be carried out in a 125 mL autoclave (model 4748, used with 33 mL of total solvents. Yield: 791 mg, ~82%) and characterized consistently with the mentioned techniques.

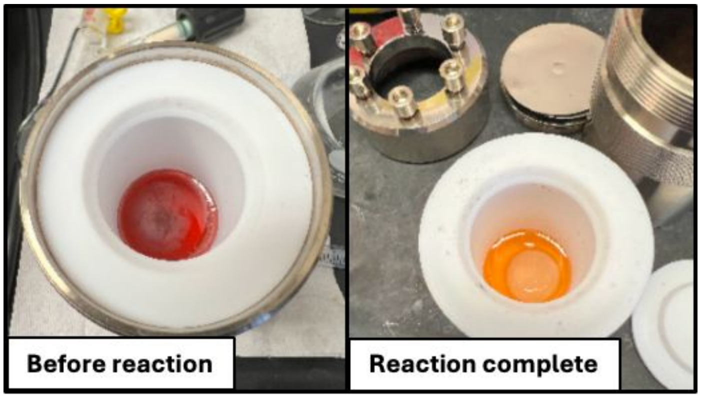

Figure S2. Images of the Teflon vessel before placing it in the oven to initiate the solvothermal reaction (on the left) and after reaction completion (on the right). The two phases benzyl alcohol/water are clearly visible with the colors orange and off-white (where the MOF is) respectively.

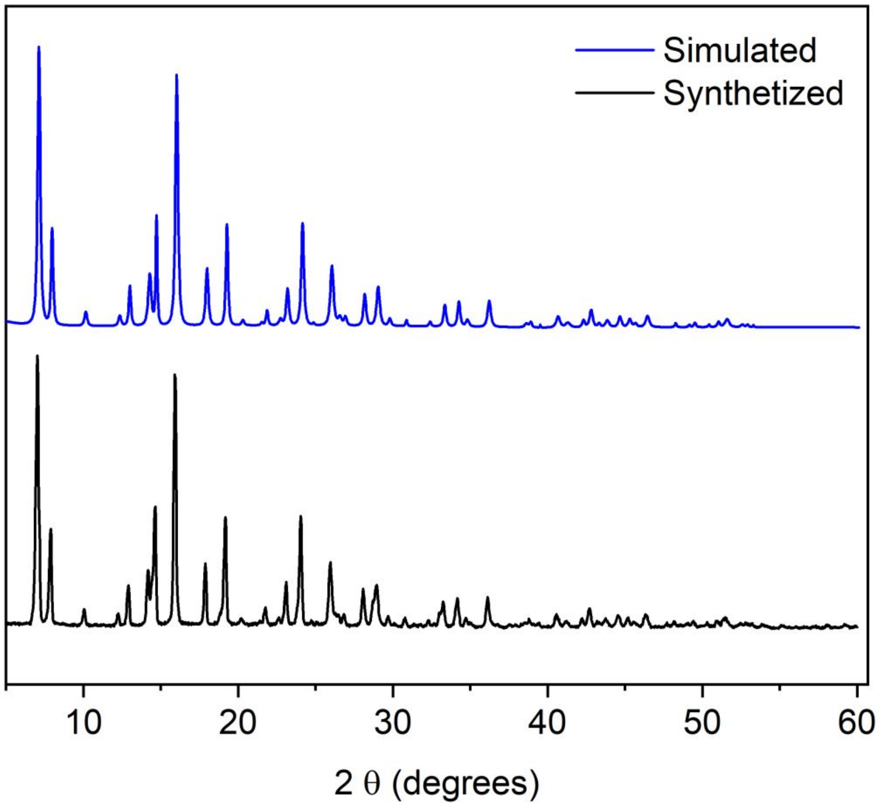

Figure S3. PXRD diffractograms of MIP-213 comparing the one obtained from the synthetized material to a simulated curve.

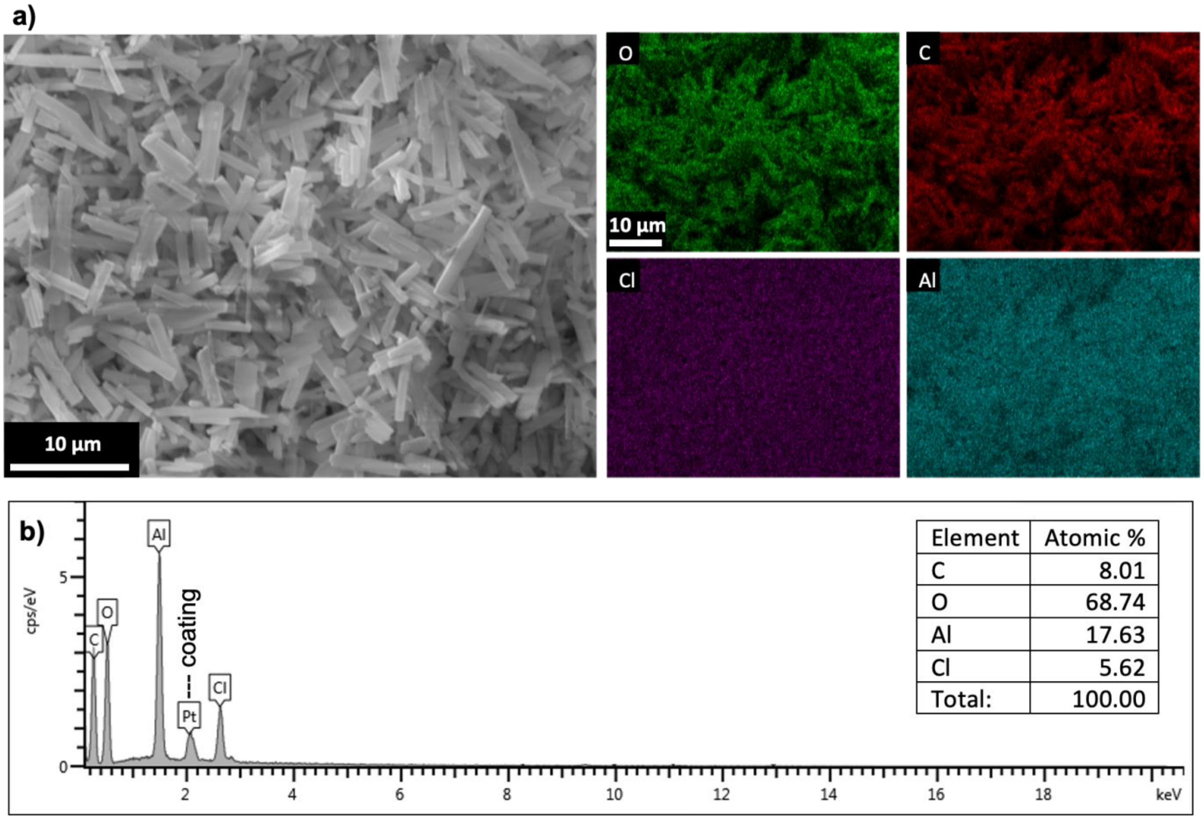

Figure S4 a) SEM image (left) and EDX map of MIP-213 (right). Color code: oxygen (O) in green, carbon (C) in red, chlorine (Cl) in purple and aluminum (Al) in turquoise; b) EDX spectrum reporting all the expected elements with rational ratio.

^{1}H-NMR performed after digestion of the MOF with sodium deuteroxide (NaOD), shows the signals relative to the linker. If present, a peak for the -CH_2 group of benzyl alcohol (solvent used for the synthesis) is expected around ~4.4 ppm; however, in the presence of NaOD, deprotonation leads to the formation of benzyl alkoxide, the -CH_2 peak of which is shifted to lower frequencies. This is observed in the spectrum as impurity (<8%) at 3.65 ppm (relative aromatic signal present as multiplet at 6.5 ppm) (**Figure S5**). ^{1}H NMR (600 MHz, D_2O, δ): 7.39 (s, 2H, Ar H), 7.05 (s, 2H, Ar H), 3.28 (s, 2H; CH_2).

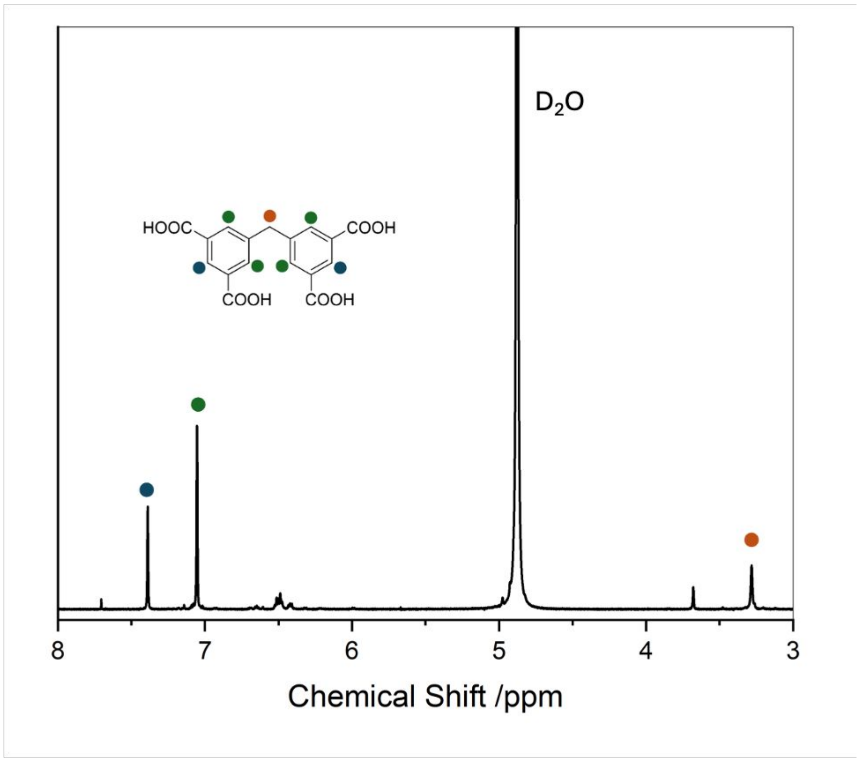

Figure S5. ^{1}H-NMR showing the spectrum of the solute obtained after digestion of the MOF with a NaOD solution.

Synthesis of FeOCl and preparation of FeOCl electrodes

Iron oxychloride (FeOCl) was synthetized via thermal decomposition of $FeCl_3 \times 6H_2O$ at 220 °C (as confirmed by XRD in **Figure S13a**), as per a reported procedure.[2] The FeOCl-based composite positive electrodes were prepared within an environmentally controlled Ar-atmosphere dry glovebox (MBraun UNILab, H_2O and O_2 content <1 ppm) from a slurry containing a 60:30:10 ratio of FeOCl:C65:PVdF accounting for ~18 wt% in a NMP solution. The slurry was deposited onto a Ni current collector foil by means of a semi-automated doctor blade coater. The solvent was removed by drying the casted electrode in the glovebox atmosphere for 3 days. Eventually, the FeOCl cathode tapes were cut into disks, dried at 120 °C under vacuum for 24 h and stored in the dry glovebox before their assembly and testing in lab-scale electrochemical test cells.

Methods

Textural and physical chemical characterization of MIP-213

Powder X-ray diffraction (PXRD) measurements were performed using a PANalytical X'Pert diffractometer (Cu Ka radiation), in Bragg-Brentano geometry, with a flat sample analyzed in open-air conditions both for the analyses post synthesis and for the screening of MIP-213 at different temperatures. In this regard, the samples were thermally treated in an oven for 2h and subsequently analyzed ex situ.

Scanning electron microscopy (SEM) was performed using a Zeiss Merlin system equipped with an energy-dispersive X-ray spectroscopy (EDS) detector. The analysis was conducted at an accelerating voltage of 20 keV after having coated the sample with few nm of a Pt layer. Thermogravimetric analysis (TGA) was performed with a TA Q600 instrument at a heating rate of 5 °C min^{-1} in a temperature range between 30 and 800 °C, under synthetic air (50 mL min^{-1}). Diffuse reflection FT-IR (DRIFT) spectroscopy was conducted on a Bruker Invenio IR spectrometer equipped with a MCT detector at 2 cm^{-1} resolution, in a temperature range between 25 and 280 °C. Sorption measurements with N_2 and CO_2 were performed using a Micromeritics 3Flex sorption analyzer at both 77 and 273 K, respectively. Before the analysis, samples (~40 mg) were weighed and treated under a high vacuum and temperature overnight. The NMR spectra were recorded on a Jeol ECZ-R 600 MHz instrument.

Ionic conductivity

The ionic conductivity (σ) of MIP-213 samples was assessed by electrochemical impedance spectroscopy (EIS) (frequency range 500 kHz – 100 mHz, with an AC voltage V_{AC} = 20 mV) on a VMP3 workstation (Biologic), using ion-blocking stainless steel (SS) electrodes in a symmetric SS|electrolyte|SS configuration, using 14 mm round pressure cells (area = 1.54 cm^2) for solid-state

assembly by Chiltern Connections LTD (Wallingford, United Kingdom). A 1/4 inches Square Drive Electronic Torque Wrench (1- 20 Nm) was used to apply a force of 10 Nm to each measured cell.

The impedance spectra (**Figure S6**) were fitted (fit parameters in Table S1) using EC-Lab V11.61 software, with the Randomize + Levenberg-Marquardt method, stopping both randomize and fit on 10 000 iterations. To fit our data, we selected a classical equivalent circuit R_1+Q_2/R_2+Q_3 + W.[3] Here R_1 represents the series resistance associated with the cell case, cables, and electrical connections, R_2 represents the bulk resistance of the whole electrochemical system (R_b), Q_2 represents a constant phase element associated with the geometrical capacitance of the cell (*i.e.* the capacitance between the electrodes, which is function of the cell geometry and electrolyte dielectric properties). Eventually Q_3 is due to capacitive contribution of charges accumulated at electrolyte/ion-blocking electrode interface,[4] and the W element represents the Warburg-type impedance linked to the semi-infinite ion diffusion. Generally, constant-phase elements (Q_i) were used instead of pure capacitive elements (C_i) to account for surface irregularities and roughness. Subsequently, σ values were calculated using Equation 1:

$$\sigma = \frac{l}{R_b A} \quad (1)$$

where l represents the thickness of the electrolyte pellet, A the contact area of the electrolyte and R_b derived from EIS.

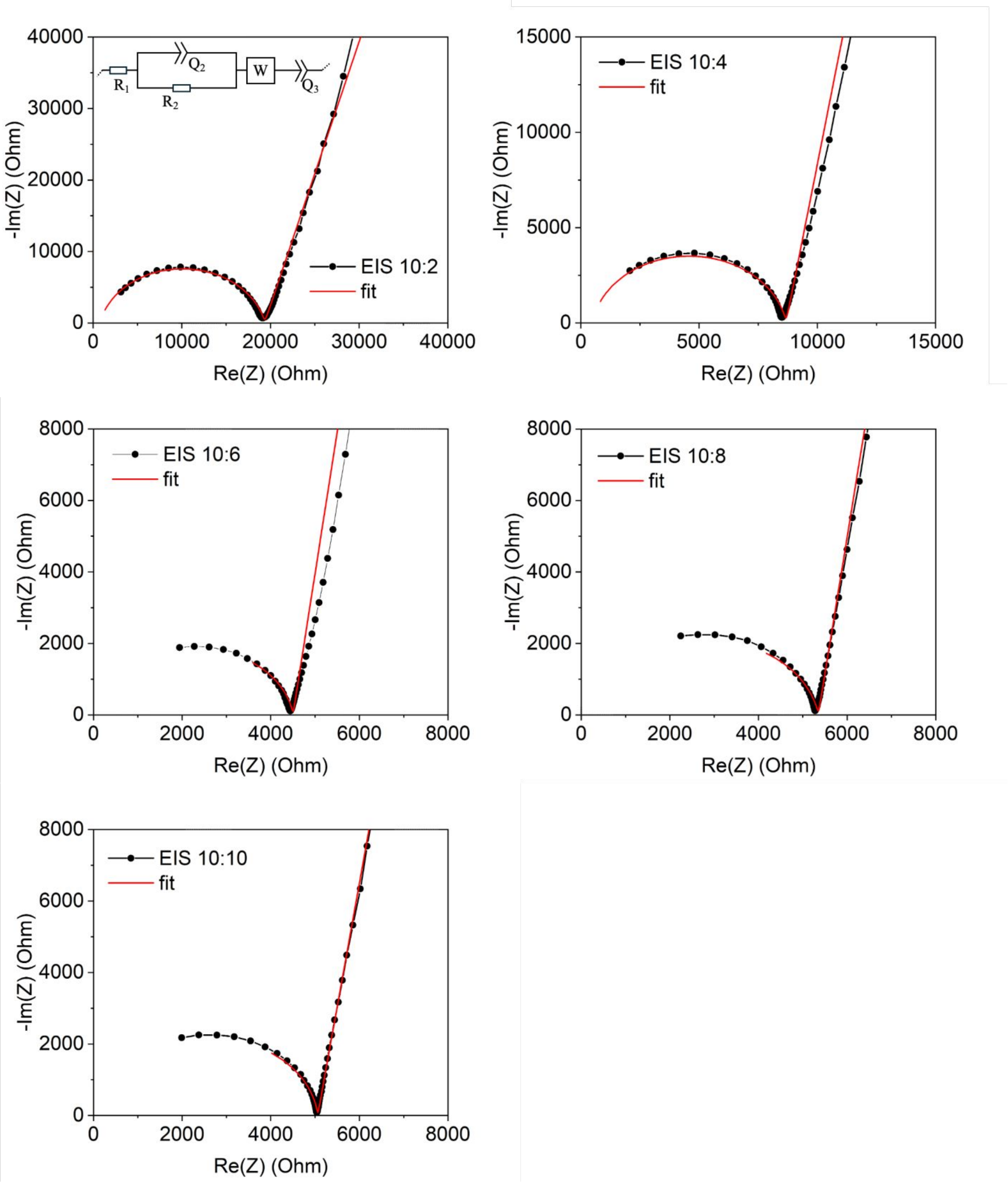

Figure S6. EIS data for the different MOF-PC mixtures explored (black curves) with fit lines (red curves) obtained using the equivalent circuit described above and reported on the upper left corner of the current figure.

Table S1 Equivalent circuit fitting parameters for the MOF-PC mixtures. R = resistance, Q = constant phase element, a = Q exponent (0–1, ideal capacitor → 1), $\chi^2/|Z|^2$ = reduced chi-squared (goodness of fit, < 0.05 indicates reliable fits).

**MOF:PC**	$\boldsymbol{R_1}$ (Ω)	$\boldsymbol{R_2}$ (kΩ)	$\boldsymbol{Q_2}$ ($F{\cdot}s^{a-1}$)	**a2**	$\boldsymbol{Q_3}$ ($F{\cdot}s^{a-1}$)	**a3**	$\boldsymbol{\chi^2/|Z|^2}$
10:2	782 [±38]	18.62 [±0.03]	5.98×10^{-10} [$\pm7.4\times10^{-12}$]	0.873 [±0.001]	3.78×10^{-6} [$\pm2.0\times10^{-8}$]	0.83 [±0.001]	0.016
10:4	480 [±20]	8.20 [±0.02]	6.09×10^{-10} [$\pm1.9\times10^{-11}$]	0.901 [±0.003]	2.11×10^{-6} [$\pm4.6\times10^{-9}$]	0.899 [±0.001]	0.023
10:6	308 [±17]	4.19 [±0.02]	5.88×10^{-10} [$\pm3.1\times10^{-11}$]	0.912 [±0.004]	3.56×10^{-6} [$\pm2.0\times10^{-8}$]	0.920 [±0.002]	0.029
10:8	552 [±37]	4.80 [±0.04]	6.38×10^{-10} [$\pm4.9\times10^{-11}$]	0.902 [±0.007]	3.435×10^{-6} [$\pm1.5\times10^{-8}$]	0.917 [±0.001]	0.023
10:10	355 [±30]	4.70 [±0.03]	3.76×10^{-10} [$\pm2.1\times10^{-11}$]	0.940 [±0.005]	5.15×10^{-6} [$\pm2.3\times10^{-8}$]	0.909 [±0.001]	0.001

The activation energy E_a was calculated using Equation 2 from the slope of the Arrhenius plot relative to the ionic conductivity data reported in the natural logarithmic scale:

$$\ln(\sigma) = \ln(\sigma_0) - \frac{E_a}{k_B}\cdot\frac{1}{T} \quad (2)$$

where σ represents ionic conductivity (in S/cm), σ_0 is the pre-exponential factor accounting for theoretical conductivity if thermal energy was infinite, E_a represents the activation energy (in eV), k_B is the Boltzmann constant (in eV K^{-1}) and T represents the absolute temperature (in K). From there, the slope is $-(E_a/k_B)$, and activation energy $E_a = -slope \times k_b$.[5–7]

Equation (3) was utilized to calculate the clamping force F applied over the area of the cell A to eventually estimate of the stack pressure ($P = F/A$) applied in this configuration.[8] This translated to ~30 MPa, which is reasonable and comparable to other studies but still above application setline (considered to be <5MPa).[9–12]

$$\mathrm{F} \approx \frac{T}{Kd} \quad (3)$$

where T is the torque (10 Nm), K is the torque coefficient (≈ 0.2 for steel lubricated bolts) and d is the nominal diameter of the bolt (0.01 m).

To understand the behavior of the MOF under different stack pressures, EIS data were measured using a MOF:PC pellet with 10:4 mass ratio (~20 mg of MOF). Using the mentioned pressure cell, each measurement was conducted on the same pellet, gradually increasing the stack pressure (**Figure S7**). Results show a significant influence of the stack pressure on conductivity which increases for higher pressure. The effect is more pronounced up to ~30 MPa, with only a slight increase on conductivity for stack pressures of ~40 to ~50 MPa.

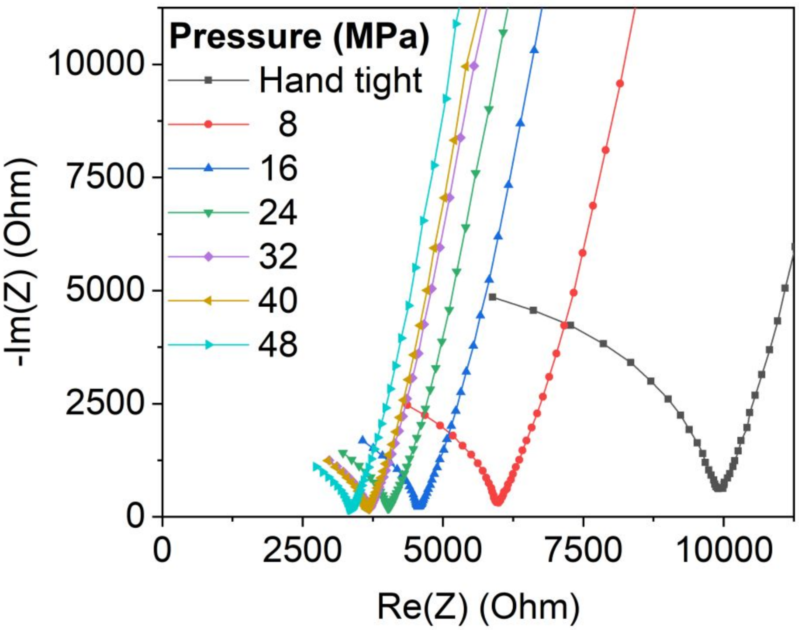

Figure S7. EIS data at different stack pressures for the mixture MOF:PC 10:4. A visible conductivity increase is noticed for values up to ~30 MPa, beyond which the effect is less prominent.

The EIS spectra to evaluate ionic conductivity under different temperatures were collected at 10 °C steps between −10 and 80 °C, inside an environmental simulation chamber (MK-53, Binder), after 1.5 h of equilibration time at each temperature. For each temperature step were

prepared four MOF pellets, each consisted of 50 mg of MOF powder mixed in a mortar till homogeneity with variable amounts of PC (ratios 10:2, 10:4, 10:6, 10:8, and 10:10). To conduct a bare-eye texture evaluation of the MOF-PC mixture, solvent aliquots were added dropwise on an analytical scale (OHAUS Explorer™ Analytical model EX12001M), mixing to homogeneity after each addition. The equivalent values, shown in amount of solvent and percentages are reported in **Table S2**.

Table S2. Aliquots of PC solvent used to assemble the cells with 50 mg of MOF.

PC (mg)	MOF:PC	wt% vs mass of MOF+PC	wt% vs mass of MOF
10	10:2	17	20
20	10:4	29	40
30	10:6	38	60
40	10:8	44	80
50	10:10	50	100

Consequently, 10:4 was chosen as the best MOF:PC ratio, since it ensured both good conductivity behavior and smooth processability (the mixture still behaves as a free-flowing powder, as noticeable in **Figure S8**).

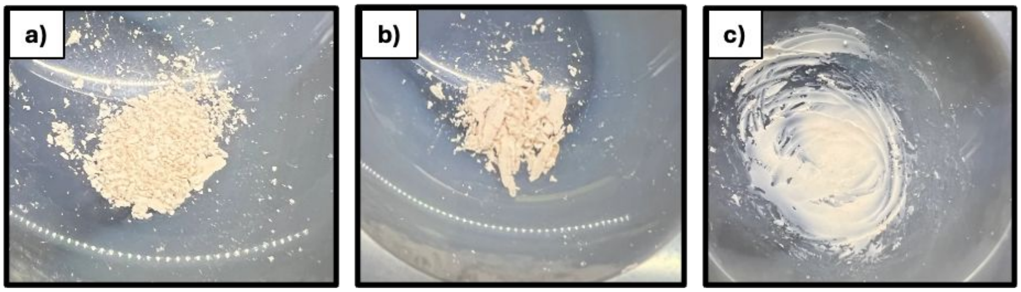

Figure S8. Photos of MIP-213 with different solvent load showing the texture change going from a) free-flowing powder using 10:4 as MOF:PC ratio; b) to sticky agglomerates using ratio 10:6; c) and eventually to a paste for the 10:10 mixture.

Note on the measurement of Cl⁻ transference number

While the Bruce–Vincent–Evans method is routinely applied to measure cation transference numbers in Li- and Na-based electrolytes, its adaptation to anion conductors is far less established. To the best of our knowledge, only Xu *et al.* have reported a first attempt to estimate a Cl^- transference number, using an Ag/AgCl symmetric cell with a liquid electrolyte.[13] Their work demonstrates feasibility but also illustrates the significant challenges (electrode preparation and stability during the measurement, long steady-state times, data reproducibility) inherent to this approach.

Electrochemical characterization

FeOCl cathodes were used as disks (Ø 14 mm) with an area (A) of 1.54 cm^2, which were cut using a high–precision punching tool EL-cut (EL-Cell Gmbh). The Li|MIP-213|FeOCl system was assembled in the same pressure cells described before (Chiltern Connections LTD, Wallingford, United Kingdom) inside a glove-box.

The electrochemical stability window and the redox processes were evaluated by cyclic voltammetry (CV) and linear sweep voltammetry (LSV) at 25 °C using Li|MIP-213|FeOCl and Li|MIP-213|Ni-CC cell configurations respectively (Ni-CC stands for carbon-coated nickel current collector). For both measurements we used a scan rate of 0.1 mV s^{-1} using a VMP3 workstation (Biologic) in the range 1.5–3.6 V vs Li^+/Li for the CV and up to 6 V vs Li^+/Li for the LSV.

For investigating the electrochemical performance, the cells Li|MIP-213|FeOCl were cycled at ambient temperature under a constant current (CC) regime with an Arbin BT2000 battery tester. The chosen current density was 15 mA g^{-1}, that correspond to a theoretical C/20 regime (i.e., a full

charge/discharge in 20 hours), based on the theoretical specific capacity of the cathode. For FeOCl it was calculated to be 250 mAh g^{-1} (Equation 4).[14]

$$C_{theoretical} = \frac{n \times F}{M} \tag{4}$$

where n refers to the number of electrons involved in the process, F is the Faraday constant and M the molecular mass of the electrode active material. From here, considering Equation 5, we calculated a theoretical specific energy density on an active material basis of ~650 Wh kg^{-1}.

$$E_{theoretical} = C_{th} \times V_{av} \tag{5}$$

where C_{th} refers to the theoretical specific capacity of the cathode and V_{av} is the average discharge potential (~2.6 V). However, we note that our present system is not optimized for power capability. The ionic conductivity of MIP-213-PC (~10^{-6} S cm^{-1} at 25 °C) indeed limits rate performance, so the practical energy and power densities of our laboratory-scale cells are expected to be lower.

Additional Results and Discussion

Table S1. A benchmark list reporting most recent results in the field of (Q)SSE plus a standard Li-ion liquid electrolyte. The table includes four Li^+ and four Cl^- conductors.

**Electrolyte**	**Mobile ion**	**Form**	**σ (25 °C) [S cm⁻¹]**	**Notes / source**
**Li_2ZrCl_6 (LZC)**	Li^+	Solid halide SSE	**8.1×10^{-4}** (as-milled); **5.8×10^{-6}** after 350 °C anneal	Nature Communications report on LZC; conductivity strongly processing-dependent.[15]
**Li_3InCl_6 (LIC)**	Li^+	Solid halide SSE	**1.5×10^{-3}**	Recent LIC with high RT conductivity.[16]
**Li_6PS_5Cl (LPSC, argyrodite)**	Li^+	Solid sulfide SSE	**2.0×10^{-3}** (typical); up to **7.3×10^{-3}** with Al/Cl tuning	Representative values across prep routes; doped compositions can reach ~7 mS cm^{-1}.[17]
**Commercial liquid (1 M $LiPF_6$ in EC:DMC 1:1)**	Li^+	Liquid	**1.2×10^{-2}**	Solvionic (France), commercial datasheet.[18]
**Polymer electrolyte for CIB**	Cl^-	Solid polymer	**10^{-5}-10^{-4}** (298-343 K)	First all-solid CIB.[2]
**Polymer electrolyte for CIB**	Cl^-	Solid polymer	**2.6×10^{-5}** (303 K)	PEG-based polymer film.[19]
**$CsSn_{0.9}In_{0.067}Cl_3$**	Cl^-	Solid halide SSE	**3.5×10^{-4}**	Halide perovskite conductor, with air processability[20]
**Cl^- membrane**	Cl^-	PVC membrane	**~10^{-7}** and **~10^{-4}**	Proof-of-concept chloride-ion membranes.[21]
**This work: MIP-213 : PC (10:4, 40 wt % PC)**	Cl^-	Quasi-solid MOF:PC composite	**1.1×10^{-6}**	Room-temperature conductivity measured in this study.

A flame-test, comparing two glass fiber (GF) disks (typically utilized as separator in liquid electrolytes-based cells) with a pellet of the MOF herein discussed is shown in **Figure S9**. To perform the experiment, the two disks are loaded with a 200 μL of different solvents. A mixture of ethylene carbonate (EC) and dimethyl carbonate (DMC) in a 1:1 ratio for the first (Figure S9, top and center panels), and just PC for the second (Figure S9 bottom panel) respectively. The MOF pellet was assembled in a pressure cell (10 Nm torque) using 25 mg of MOF powder and 10 mg of PC (MOF:PC is 10:4). Figure S9 shows progressions of the target object in three steps. Photos in column $\mathbf{t_0}$ are taken right before the flame gets in contact with the object. Column $\mathbf{t_1}$ shows photos taken upon ~1 sec of flame exposure, and column $\mathbf{t_2}$ shows photos taken ~1 sec after the flame is turned off. For the MOF pellet, no flame is observed in its last photo which demonstrates already enhanced safety or being employed as SSE. Additionally, considering the area of the objects (1.54 cm^2) the loading ratios are 129.9 and 6.5 μL cm^{-2} for GF disks and the MOF pellet, respectively. Thus, the MIP-213 pellet requires just 1/20 of the solvent to function.

Furthermore, the flame-test confirms the choice of PC as molecular transporter, a choice that took into account the different flash points of all the different carbonates typically used as solvents in batteries: negligible for EC alone being a solid; ~18 °C for DMC and ~25 °C for the 1:1 mixture of the two, while 132 °C for PC. Reasonably, both systems catch on fire if exposed to direct flame, but the latter is generally safer. Moreover, PC has a higher dielectric constant than the EC:DMC mixture (~64 and ~40, respectively), which generally favors more efficient ion solvation.

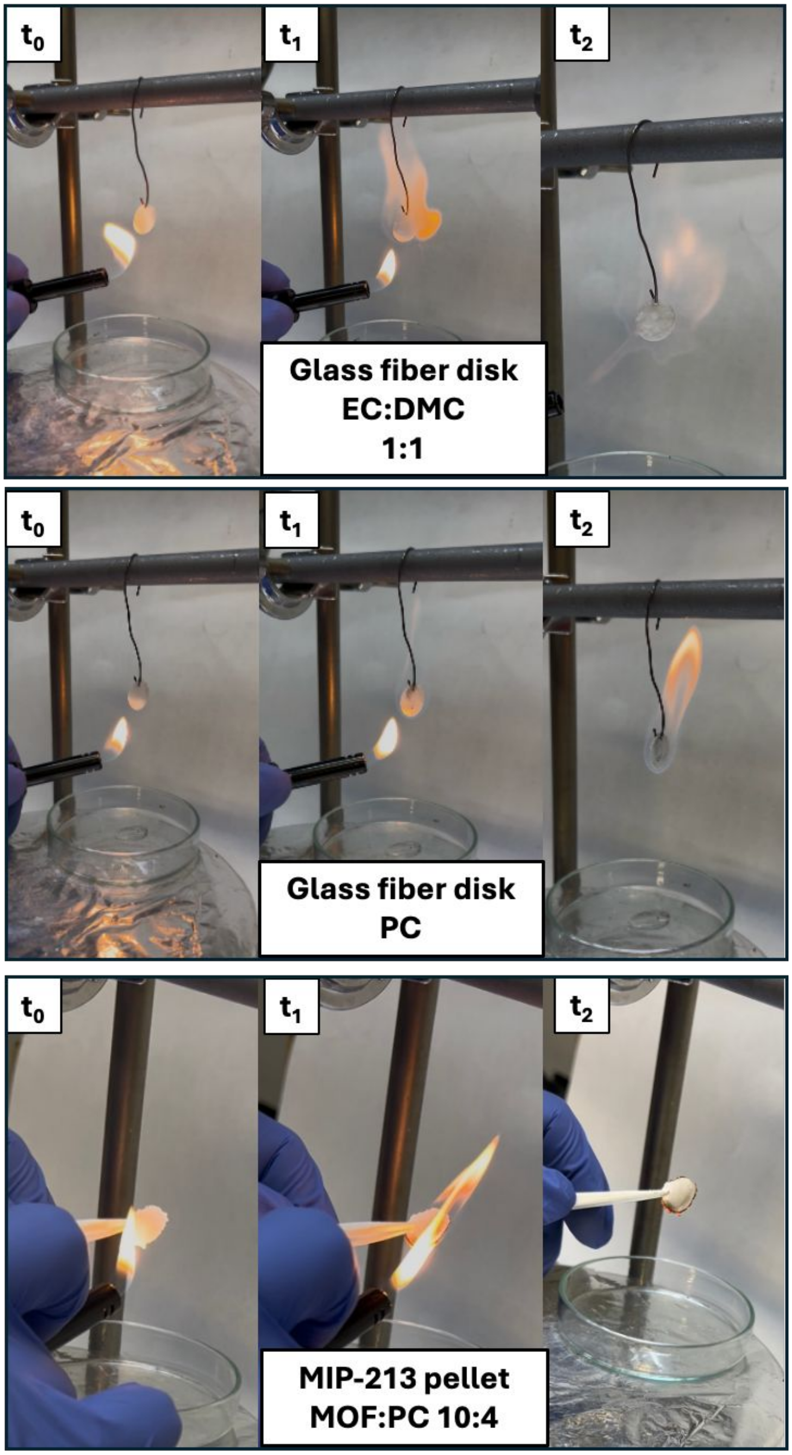

Figure S9. Flame-test comparing glass fiber disks soaked with electrolytes to a MIP-213 pellet. Where $\mathbf{t_0}$ is the instant right before the flame gets in contact with the object; $\mathbf{t_1}$ is after 1 s of flame exposure and $\mathbf{t_2}$ is 1 s after turning off the flame. Average flame exposure for all the samples is 1.5 ±0.2 s.

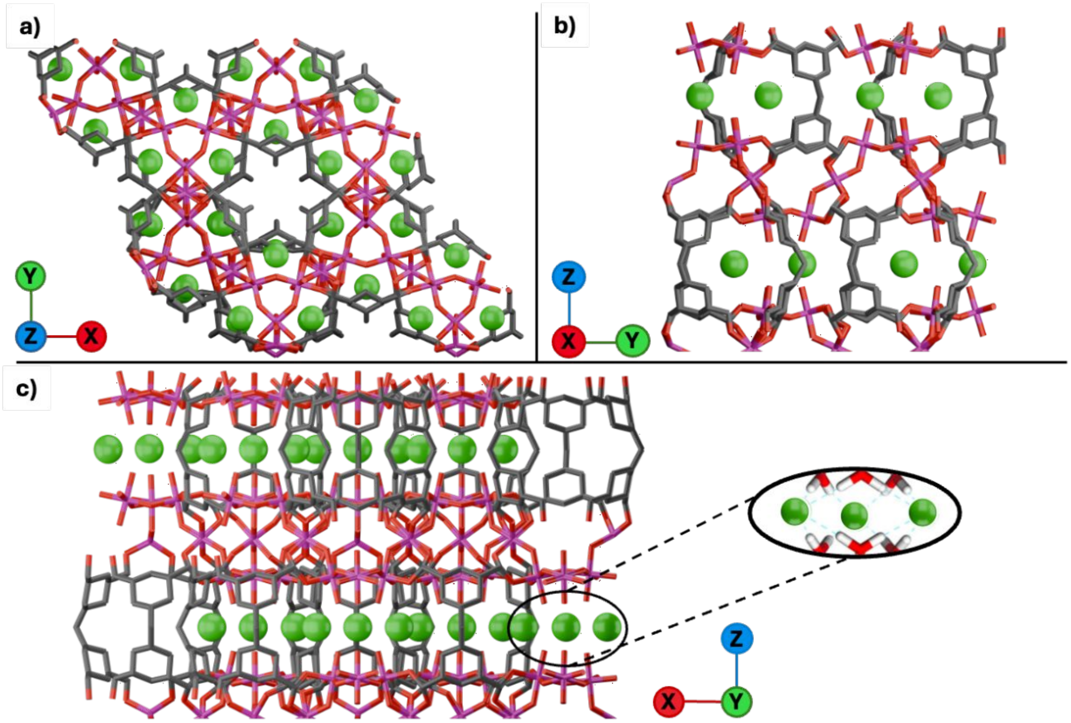

Figure S10. Overview on MIP-213 structure. a) view along the z–axis displaying the central honeycomb 18-mebered ring; b) view along the x–axis, with focus on the cavities; c) view along the y–axis, which allows an easier visualization of Cl^- ions sandwiched between two Al-trimers. Color code: aluminum, carbon, oxygen, and chloride atoms are in magenta, gray, red, and green, respectively. (hydrogen atoms are omitted for clarity). Reproduced using the CIF file published by Nandi et al., originally published under a CC BY 4.0 license.[1]

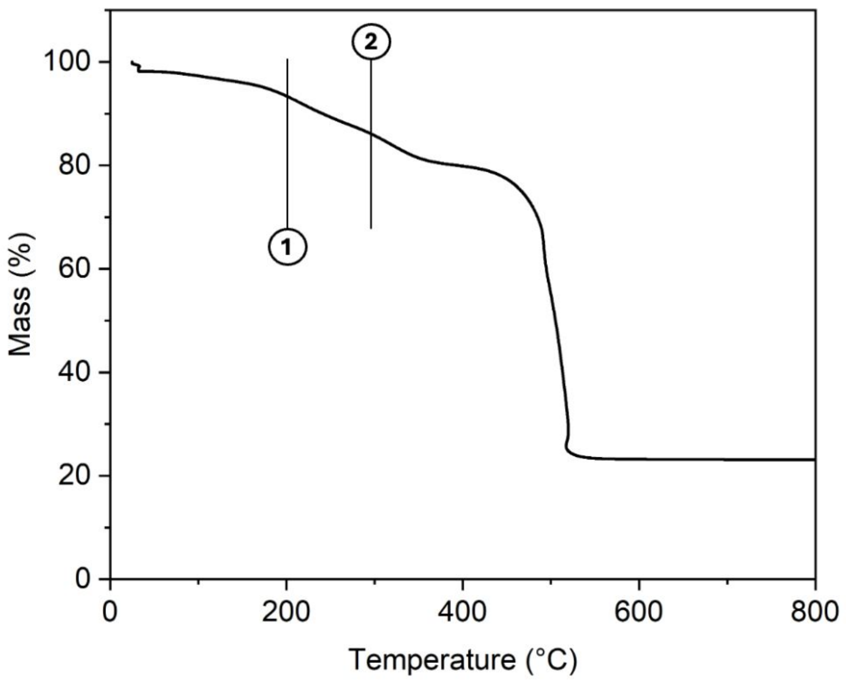

Figure S11. TGA profile of MIP-213 in air.

A clear type I isotherm, as expected for micro-porous materials like MIP-213 was obtained from sorption analysis with CO_2 as probe (**Figure S12**). The small degree of sorption/desorption hysteresis can be explained by a concerted effect between the actual ultra-microporous (~4.7 Å) nature of this MOF and the presence of Cl^-, which can retain the adsorbed probe gas.[22,23] In good accordance with the reference, the activated MOF uptakes ~1.4 mmol g^{-1} of CO_2 at 0 °C.[1]

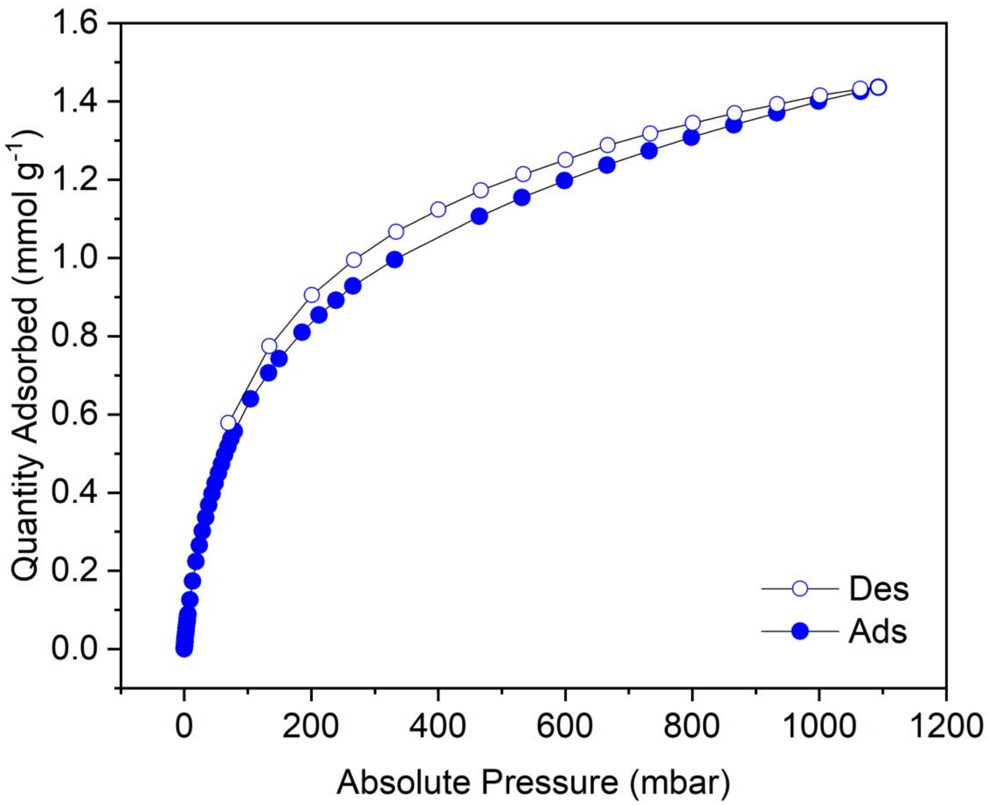

Figure S12. Isotherm showing the CO_2 adsorption profile of MIP-213 at 0 °C, after activation at 200 °C. It is in accordance with the original reference.

PXRD patterns of the cathode material before and after galvanostatic charge/discharge cycling are shown in **Figure S13**. FeOCl is expected to partially reduce to FeO, however, no diffraction peaks corresponding to FeO are observed, which suggest the formation of amorphous and/or nanosized particles. The same reason can also explain disappearance of FeOCl reflections after cycling (Figure S13b).

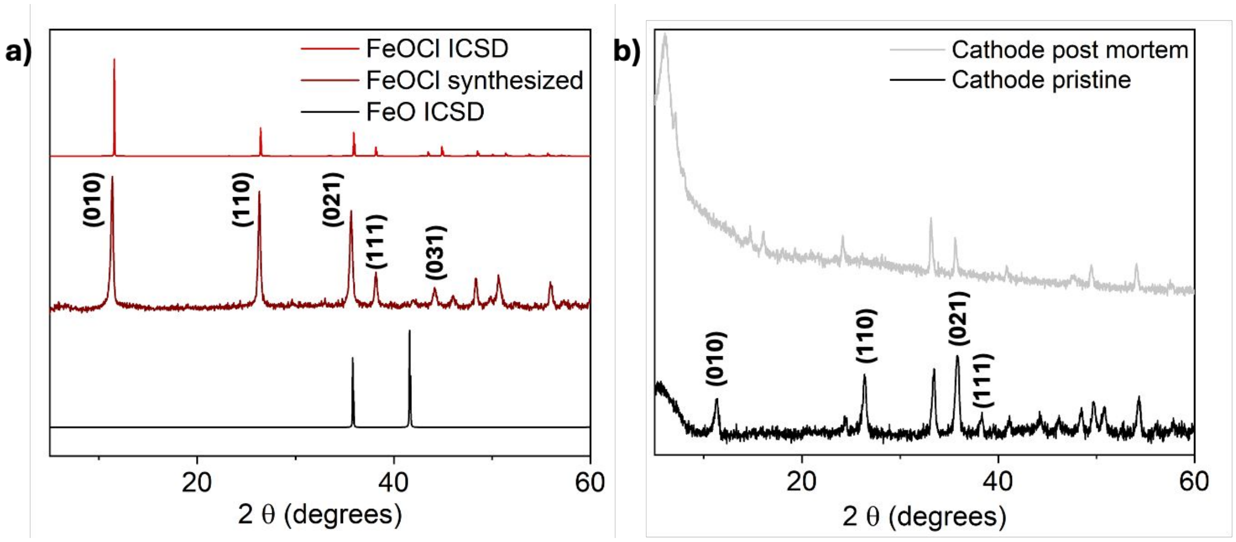

Figure S13. a) PXRD simulated patterns of FeOCl and FeO in comparison with the synthetized FeOCl. b) PXRD patterns comparing cathode materials before and after galvanostatic charge/discharge cycling.

The smooth CV profile (partially shown in Figure 3a -main text- and fully reported here in **Figure S14**) shows two different processes of oxidation at ~2.40 and ~3.25 V with respective reduction peaks at ~1.80 and ~2.55 V which could be ascribed to the FeOCl/FeO conversion, alongside with minor $FeOCl_x$ intercalation reactions.[24] These results align closely with those of FeOCl reported in the literature,[14,25–27] showing no additional features that would suggest parasitic H^+/OH^- activity.

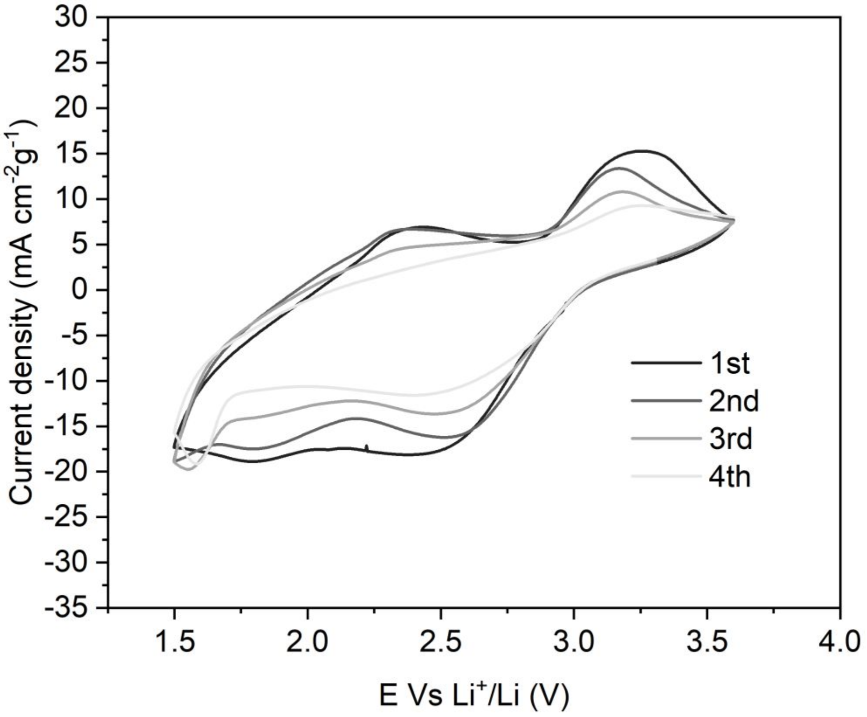

Figure S14. Cyclic Voltammetry of the cell Li|MIP-213|FeOCl, assembled with MIP-213 pellet at 10:4 MOF:PC ratio.

The voltage profile is in accordance with the CV and literature data.[2,25] Figure S14b shows the results for the second to fifth cycle plus cycles 20th and 100th, highlighting the capacity drop from an initial ~155 mAh g^{-1} to ~20 mAh g^{-1}, but also the reversible s-type profiles typical of the cathode material. Improving the cathode preparation and formulation could help to yield better results.

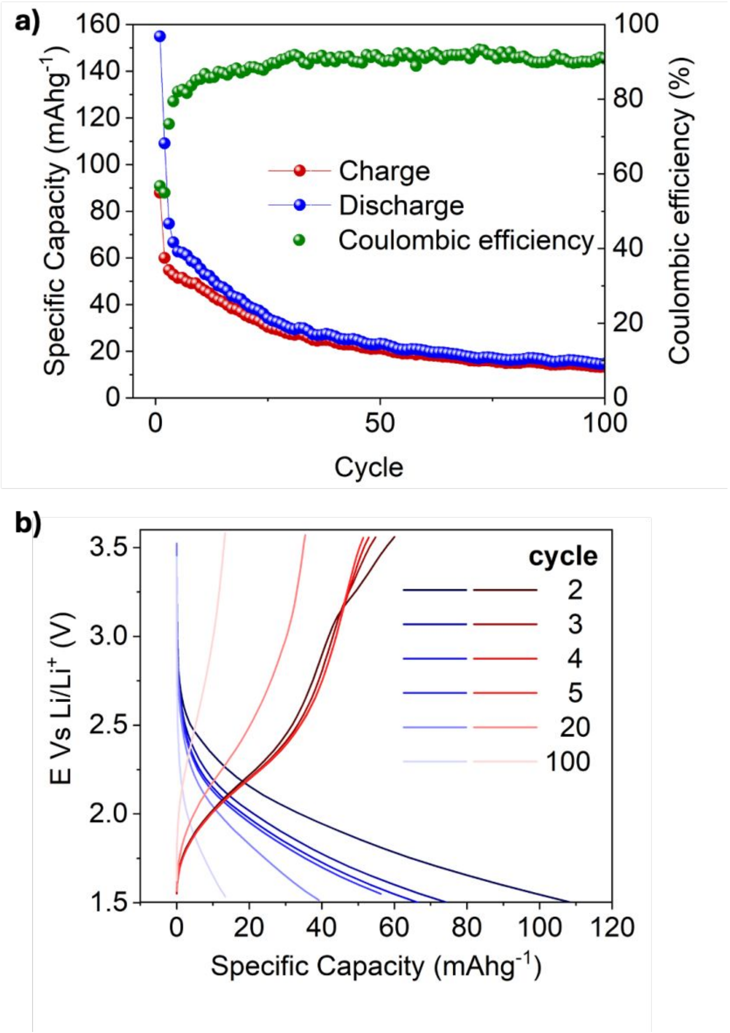

Figure S15. a) Galvanostatic charge/discharge cycling and b) potential vs specific capacity profiles of the cell Li|MIP-213|FeOCl at 25 °C, where MIP-213 is used as a pellet with 10:4 as MOF:PC ratio. Current applied: 15 mA g^{-1} (theoretical C/20 regime).

SEM-EDX analysis post cycling show that the morphology of the particles is maintained (**Figure S16a**). The higher presence of carbon and oxygen is due to presence of PC in the pores of the materials, while the lower Al:Cl ratio (1:4 here, compared to a former 1:3) (Figure S16b), reasonably caused by the irreversible processes at the cathode.

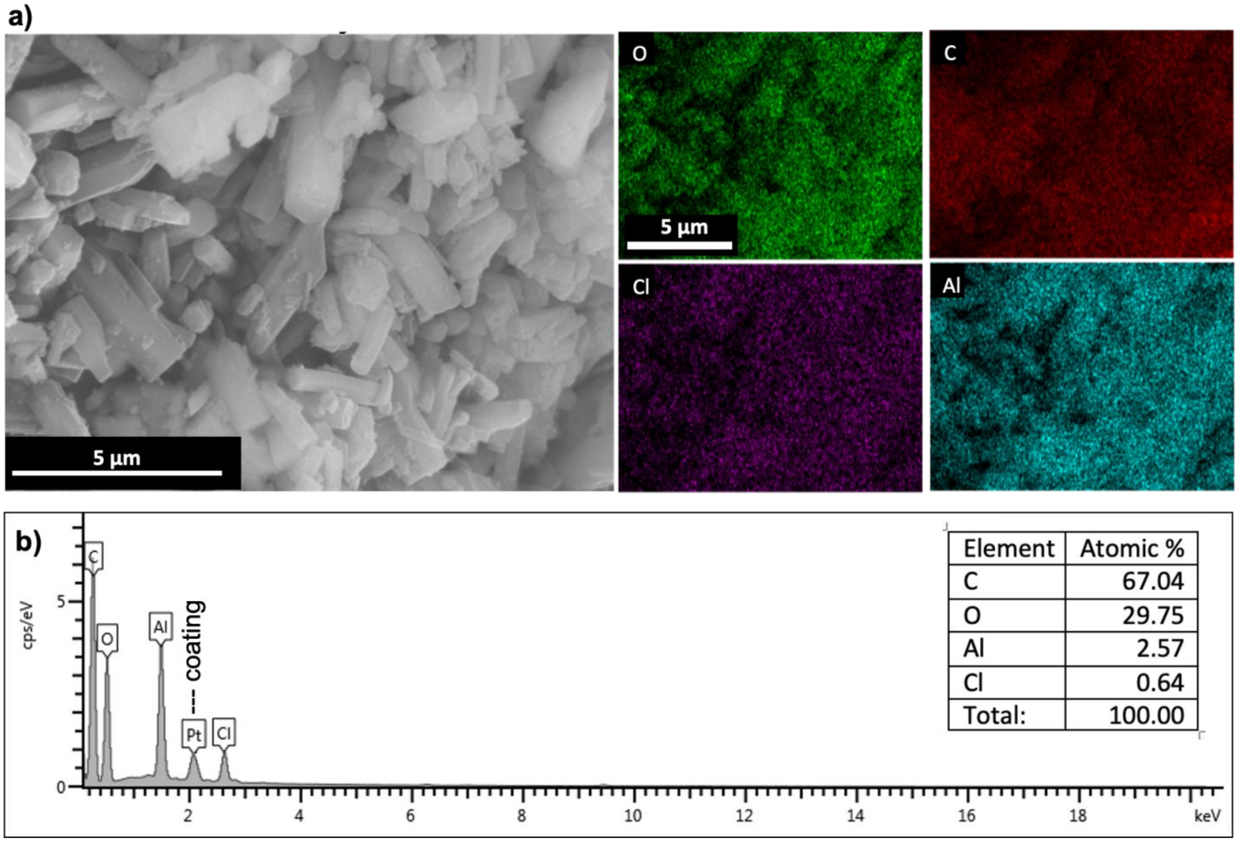

Figure S16. a) SEM image (left) and EDX map of postmortem MIP-213 (right). Color code: oxygen (O) in green, carbon (C) in red, chlorine (Cl) in purple and aluminum (Al) in turquoise; b) EDX spectrum reporting all the expected elements with rational ratio.

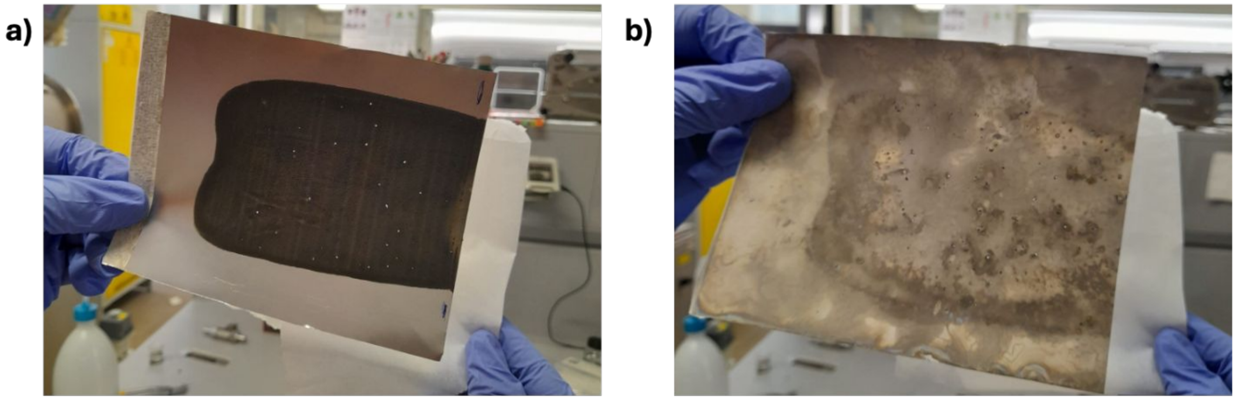

Figure S17. a) front and b) back sides of a Ni foil current collector with the FeOCl:CB:PVdF 60:30:10 casted mixture showing severe pitting corrosion consistent with evolution of HCl from FeOCl hydrolysis.

The video of flame-test is also available online as multimedia file associated with the article.

REFERENCES

(1) Nandi, S.; Mansouri, A.; Dovgaliuk, I.; Boullay, P.; Patriarche, G.; Cornu, I.; Florian, P.; Mouchaham, G.; Serre, C. A Robust Ultra-Microporous Cationic Aluminum-Based Metal-Organic Framework with a Flexible Tetra-Carboxylate Linker. *Commun Chem* **2023**, *6* (1). https://doi.org/10.1038/s42004-023-00938-x.

(2) Chen, C.; Yu, T.; Yang, M.; Zhao, X.; Shen, X. An All-Solid-State Rechargeable Chloride Ion Battery. *Advanced Science* **2019**, *6* (6). https://doi.org/10.1002/advs.201802130.

(3) Mindemark, J.; Lacey, M. J.; Bowden, T.; Brandell, D. Beyond PEO—Alternative Host Materials for Li + -Conducting Solid Polymer Electrolytes. *Prog Polym Sci* **2018**, *81*, 114–143. https://doi.org/10.1016/j.progpolymsci.2017.12.004.

(4) Orazem, M. E.; Tribollet, B. Impedance of Materials. In *Electrochemical Impedance Spectroscopy*; Wiley, 2017; pp 303–318. https://doi.org/10.1002/9781119363682.ch12.

(5) Weng, S.; Zhang, X.; Yang, G.; Zhang, S.; Ma, B.; Liu, Q.; Liu, Y.; Peng, C.; Chen, H.; Yu, H.; Fan, X.; Cheng, T.; Chen, L.; Li, Y.; Wang, Z.; Wang, X. Temperature-Dependent Interphase Formation and Li+ Transport in Lithium Metal Batteries. *Nat Commun* **2023**, *14* (1), 4474. https://doi.org/10.1038/s41467-023-40221-0.

(6) Zhao, Q.; Liu, X.; Zheng, J.; Deng, Y.; Warren, A.; Zhang, Q.; Archer, L. Designing Electrolytes with Polymerlike Glass-Forming Properties and Fast Ion Transport at Low Temperatures. *Proceedings of the National Academy of Sciences* **2020**, *117* (42), 26053–26060. https://doi.org/10.1073/pnas.2004576117.

(7) Abe, T.; Ohtsuka, M.; Sagane, F.; Iriyama, Y.; Ogumi, Z. Lithium Ion Transfer at the Interface between Lithium-Ion-Conductive Solid Crystalline Electrolyte and Polymer Electrolyte. *J Electrochem Soc* **2004**, *151* (11), A1950. https://doi.org/10.1149/1.1804813.

(8) Barrett, R. *NASA RP-1228*; 1990.

(9) Gong, Y.; Zhao, C.; Wang, D.; Wang, X.; Wang, Z.; Wu, Y.; Xia, Y.; Jing, Q.; Ji, Y.; Jiang, Y.; Liang, J.; Li, X.; Jiang, T.; Sun, X.; Zhai, X.; Sun, H.; Sun, X. Advancing High-Voltage Halide-Based Solid-State Batteries: Interfacial Challenges, Material Innovations, and Applications. *Energy Storage Mater* **2025**, *74*, 103980. https://doi.org/10.1016/j.ensm.2024.103980.

(10) Puls, S.; Nazmutdinova, E.; Kalyk, F.; Woolley, H. M.; Thomsen, J. F.; Cheng, Z.; Fauchier-Magnan, A.; Gautam, A.; Gockeln, M.; Ham, S.-Y.; Hasan, M. T.; Jeong, M.-G.; Hiraoka, D.; Kim, J. S.; Kutsch, T.; Lelotte, B.; Minnmann, P.; Miß, V.; Motohashi, K.; Nelson, D. L.; Ooms, F.; Piccolo, F.; Plank, C.; Rosner, M.; Sandoval, S. E.; Schlautmann, E.; Schuster, R.; Spencer-Jolly, D.; Sun, Y.; Vishnugopi, B. S.; Zhang, R.; Zheng, H.; Adelhelm, P.; Brezesinski, T.; Bruce, P. G.; Danzer, M.; El Kazzi, M.; Gasteiger, H.; Hatzell, K. B.; Hayashi, A.; Hippauf, F.; Janek, J.; Jung, Y. S.; McDowell, M. T.; Meng, Y. S.; Mukherjee, P. P.; Ohno, S.; Roling, B.; Sakuda, A.; Schwenzel, J.; Sun, X.; Villevieille,

C.; Wagemaker, M.; Zeier, W. G.; Vargas-Barbosa, N. M. Benchmarking the Reproducibility of All-Solid-State Battery Cell Performance. *Nat Energy* **2024**, *9* (10), 1310–1320. https://doi.org/10.1038/s41560-024-01634-3.

(11) Sang, J.; Tang, B.; Qiu, Y.; Fang, Y.; Pan, K.; Zhou, Z. How Does Stacking Pressure Affect the Performance of Solid Electrolytes and All-Solid-State Lithium Metal Batteries? *ENERGY & ENVIRONMENTAL MATERIALS* **2024**, *7* (4). https://doi.org/10.1002/eem2.12670.

(12) Doux, J.; Nguyen, H.; Tan, D. H. S.; Banerjee, A.; Wang, X.; Wu, E. A.; Jo, C.; Yang, H.; Meng, Y. S. Stack Pressure Considerations for Room-Temperature All-Solid-State Lithium Metal Batteries. *Adv Energy Mater* **2020**, *10* (1). https://doi.org/10.1002/aenm.201903253.

(13) Xu, C.; Lei, C.; Li, J.; He, X.; Jiang, P.; Wang, H.; Liu, T.; Liang, X. Unravelling Rechargeable Zinc-Copper Batteries by a Chloride Shuttle in a Biphasic Electrolyte. *Nat Commun* **2023**, *14* (1), 2349. https://doi.org/10.1038/s41467-023-37642-2.

(14) Zhao, X.; Li, Q.; Yu, T.; Yang, M.; Fink, K.; Shen, X. Carbon Incorporation Effects and Reaction Mechanism of FeOCl Cathode Materials for Chloride Ion Batteries. *Sci Rep* **2016**, *6* (1), 19448. https://doi.org/10.1038/srep19448.

(15) Wang, K.; Ren, Q.; Gu, Z.; Duan, C.; Wang, J.; Zhu, F.; Fu, Y.; Hao, J.; Zhu, J.; He, L.; Wang, C.-W.; Lu, Y.; Ma, J.; Ma, C. A Cost-Effective and Humidity-Tolerant Chloride Solid Electrolyte for Lithium Batteries. *Nat Commun* **2021**, *12* (1), 4410. https://doi.org/10.1038/s41467-021-24697-2.

(16) Jin, F.; Fadillah, L.; Nguyen, H. Q.; Sandvik, T. M.; Liu, Y.; García-Martín, A.; Salagre, E.; Michel, E. G.; Stoian, D.; Marshall, K.; Van Beek, W.; Redhammer, G.; Mehraj Ud Din, M.; Rettenwander, D. Elucidating the Impact of Li_3InCl_6-Coated $LiNi_{0.8}Co_{0.15}Al_{0.05}O_2$ on the Electro-Chemo-Mechanics of Li_6PS_5Cl-Based Solid-State Batteries. *Chemistry of Materials* **2024**, *36* (12), 6017–6026. https://doi.org/10.1021/acs.chemmater.4c00515.

(17) Lee, J. M.; Park, Y. S.; Moon, J.-W.; Hwang, H. Ionic and Electronic Conductivities of Lithium Argyrodite Li6PS5Cl Electrolytes Prepared via Wet Milling and Post-Annealing. *Front Chem* **2021**, *9*. https://doi.org/10.3389/fchem.2021.778057.

(18) https://solvionic.com/en/electrolytes/5607-1m-lipf6-in-dmc-ec-1-1-vol.html?utm. *E001 1M LIPF6 IN DMC:EC 1:1 (VOL.)*.

(19) Wang, Q.; Liu, J.; Zhang, J.; Lou, X.-M.; Tan, Q.; Gao, P. A Polymer Electrolyte for Rechargeable Chloride Ion Batteries. *Mater Lett* **2022**, *329*, 133150. https://doi.org/10.1016/j.matlet.2022.133150.

(20) Karkera, G.; Soans, M.; Akbaş, A.; Witter, R.; Euchner, H.; Diemant, T.; Cambaz, M. A.; Meng, Z.; Dasari, B.; Chandrappa, S. G.; Menezes, P. W.; Fichtner, M. A Structurally Flexible Halide Solid Electrolyte with High Ionic Conductivity and Air Processability. *Adv Energy Mater* **2023**, *13* (30). https://doi.org/10.1002/aenm.202300982.

(21) Gschwind, F.; Steinle, D.; Sandbeck, D.; Schmidt, C.; von Hauff, E. Facile Preparation of Chloride-Conducting Membranes: First Step towards a Room-Temperature Solid-State Chloride-Ion Battery. *ChemistryOpen* **2016**, *5* (6), 525–530. https://doi.org/10.1002/open.201600109.

(22) Sapchenko, S. A.; Barsukova, M. O.; Belosludov, R. V.; Kovalenko, K. A.; Samsonenko, D. G.; Poryvaev, A. S.; Sheveleva, A. M.; Fedin, M. V.; Bogomyakov, A. S.; Dybtsev, D. N.; Schröder, M.; Fedin, V. P. Understanding Hysteresis in Carbon Dioxide Sorption in Porous Metal–Organic Frameworks. *Inorg Chem* **2019**, *58* (10), 6811–6820. https://doi.org/10.1021/acs.inorgchem.9b00016.

(23) Wang, S.; Yang, Q.; Zhang, J.; Zhang, X.; Zhao, C.; Jiang, L.; Su, C.-Y. Two-Dimensional Charge-Separated Metal–Organic Framework for Hysteretic and Modulated Sorption. *Inorg Chem* **2013**, *52* (8), 4198–4204. https://doi.org/10.1021/ic301781n.

(24) Zhao, X.; Zhao-Karger, Z.; Wang, D.; Fichtner, M. Metal Oxychlorides as Cathode Materials for Chloride Ion Batteries. *Angewandte Chemie International Edition* **2013**, *52* (51), 13621–13624. https://doi.org/10.1002/anie.201307314.

(25) Yu, T.; Zhao, X.; Ma, L.; Shen, X. Intercalation and Electrochemical Behaviors of Layered FeOCl Cathode Material in Chloride Ion Battery. *Mater Res Bull* **2017**, *96*, 485–490. https://doi.org/10.1016/j.materresbull.2017.03.070.

(26) Yu, T.; Li, Q.; Zhao, X.; Xia, H.; Ma, L.; Wang, J.; Meng, Y. S.; Shen, X. Nanoconfined Iron Oxychloride Material as a High-Performance Cathode for Rechargeable Chloride Ion Batteries. *ACS Energy Lett* **2017**, *2* (10), 2341–2348. https://doi.org/10.1021/acsenergylett.7b00699.

(27) Zhang, C.; Sun, S.; Wu, M.; Zhao, X. FeOCl Nanoparticle-Embedded Mesocellular Carbon Foam as a Cathode Material with Improved Electrochemical Performance for Chloride-Ion Batteries. *ACS Appl Mater Interfaces* **2023**, *15* (4), 5209–5217. https://doi.org/10.1021/acsami.2c19299.
